# Intranasal Administration of Extracellular Vesicles Derived from Adipose Mesenchymal Stem Cells Has Therapeutic Effect in Experimental Autoimmune Encephalomyelitis

**DOI:** 10.3390/cells14151172

**Published:** 2025-07-30

**Authors:** Barbara Rossi, Federica Virla, Gabriele Angelini, Ilaria Scambi, Alessandro Bani, Giulia Marostica, Mauro Caprioli, Daniela Anni, Roberto Furlan, Pasquina Marzola, Raffaella Mariotti, Gabriela Constantin, Bruno Bonetti, Ermanna Turano

**Affiliations:** 1 Division of General Pathology, Department of Medicine, University of Verona, 37134 Verona, Italy; gabriele.angelini@univr.it (G.A.); alessandro.bani@univr.it (A.B.); gabriela.constantin@univr.it (G.C.); 2 Department of Neuroscience, Biomedicine and Movement Sciences, University of Verona, 37134 Verona, Italy; federica.virla@univr.it (F.V.); ilaria.scambi@univr.it (I.S.); mauro.caprioli@univr.it (M.C.); daniela.anni@univr.it (D.A.); raffaella.mariotti@univr.it (R.M.); 3 Clinical Neuroimmunology Unit, Institute of Experimental Neurology, San Raffaele Scientific Institute, 20132 Milan, Italy; giulia.marostica@hotmail.it (G.M.); furlan.roberto@hsr.it (R.F.); 4 Department of Engineering for Innovation Medicine, University of Verona, 37134 Verona, Italy; pasquina.marzola@univr.it; 5 Neurology Unit, Azienda Ospedaliera Universitaria Integrata Verona, 37126 Verona, Italy; bruno.bonetti@aovr.veneto.it

**Keywords:** extracellular vesicles, multiple sclerosis, EAE, stem cells, adipose mesenchymal stem cells

## Abstract

Adipose stem cells (ASCs) are a subset of mesenchymal stem cells with validated immunomodulatory and regenerative capabilities that make them attractive tools for treating neurodegenerative disorders, such as multiple sclerosis (MS). Several studies conducted on experimental autoimmune encephalomyelitis (EAE), the animal model of MS, have clearly shown a therapeutic effect of ASCs. However, controversial data on their efficacy were obtained from I- and II-phase clinical trials in MS patients, highlighting standardization issues and limited data on long-term safety. In this context, ASC-derived extracellular vesicles from (ASC-EVs) represent a safer, more reproducible alternative for EAE and MS treatment. Moreover, their physical characteristics lend themselves to a non-invasive, efficient, and easy handling of intranasal delivery. Using an in vitro setting, we first verified ASC-EVs’ ability to cross the human nasal epithelium under an inflammatory milieu. Magnetic resonance corroborated these data in vivo in intranasally treated MOG35-55-induced EAE mice, showing a preferential accumulation of ASC-EVs in brain-inflamed lesions compared to a stochastic distribution in healthy control mice. Moreover, intranasal treatment of ASC-EVs at the EAE onset led to a long-term therapeutic effect using two different experimental protocols. A marked reduction in T cell infiltration, demyelination, axonal damage, and cytokine production were correlated to EAE amelioration in ASC-EV-treated mice compared to control mice, highlighting the immunomodulatory and neuroprotective roles exerted by ASC-EVs during EAE progression. Overall, our study paves the way for promising clinical applications of self-administered ASC-EV intranasal treatment in CNS disorders, including MS.

## 1. Introduction

Adult mesenchymal stem cells (MSCs) represent a potential therapy for severe chronic neurological diseases [1] and may provide an alternative therapy to cure multiple sclerosis (MS). The use of MSCs is supported by decades of in vitro and in vivo preclinical studies showing their immunomodulatory and neuroprotective properties [2,3,4,5], encouraging many clinical studies. Several clinical trials have investigated the application of MSC therapies in MS, globally validating the safety and tolerability of MSC transplantation [6,7,8]. Optimistic results were reported by Petrou and colleagues in progressive MS patients in a randomized, double-blind, placebo-controlled trial [9]. However, less enthusiastic data were obtained in the MEsenchyml StEm cells for Multiple Sclerosis (MESEMS) study, the most significant randomized double-blind phase 2 study in MS [10]. The experimenters, while confirming the safety of the treatment with modest effects on relapses, showed no effect on the primary outcome of the study, such as the number of gadolinium-enhancing lesions (GELs) at 24 weeks in the group of patients with active forms of the disease [11]. The study has highlighted several critical points that may explain the lack of therapeutic activity of MSCs, ranging from the heterogeneity of enrolled patients to technical issues, including the lack of uniformity of MSC sources, the route of administration, and the MSC dosage. In addition, no outcomes related to the, more specifically, neurodegenerative effects were evaluated [10].

The gap that persists in translating the promising preclinical results into a future real clinical practice is a challenge that requires standardization of protocols for in vitro cell expansion and storage, further advancement in understanding stem cell biology, defining therapeutic targets and the mode of MSC transplantation [12,13]. Starting from the biological basis, it is now known that mesenchymal stem cells exert their effects not through direct cell replacement of damaged tissues but rather through the secretion of a plethora of trophic and immunomodulatory factors released mainly in extracellular vesicles (EVs) whose composition varies according to the tissue of origin and culture conditions [14,15,16]. In this regard, using MSC-EVs represents a potential alternative to the parental cells, demonstrating the same efficacy, avoiding many risks associated with cell transplantation, and offering advantages in production and handling processes [17,18]. It is indeed well established that MSC-EVs maintain and exert the same functions wielded by the cells from which they derive, including immunomodulation and neuroprotection [19,20,21,22,23,24]. We previously demonstrated how preventive intravenous (i.v.) administration of small EVs derived from adipose mesenchymal stem cells (ASC-EVs) could ameliorate experimental autoimmune encephalomyelitis (EAE), the animal model of MS, by interfering mainly with peripheral immune activation and autoreactive T lymphocyte recruitment into CNS [20]. Interestingly, i.v. injection of USPIO-labelled ASC-EVs in EAE-induced mice has confirmed lymph nodes as the primary target organ these vesicles reach without evidence of iron accumulation in the CNS [25]. These data clearly emphasize a primary immunomodulatory impact that ASC-EVs exert on EAE pathogenesis when systemically injected.

To gain insights into ASC-EVs’ neuroprotective role, we evaluated their intranasal (i.n.) delivery as a potential therapeutic protocol for EAE. The i.n. delivery route takes advantage of the direct nose-to-brain connection to circumvent the BBB and deliver drugs directly to the CNS. In this paper, we first in vitro ranked ASC-EVs’ capacity to diffuse across a human nasal epithelium. Then, we validate the in vivo efficacy outcomes of intranasal treatment of ASC-EVs in the chronic EAE model, which may promote and support the development of new therapies to counteract several pathological aspects of MS. Moreover, non-invasiveness, ease of administration, fast action, and reduced side effects make the intranasal route a promising strategy to minimize the risk–benefit ratio and afford compliance in patients affected by neurological disorders, including MS [26,27,28].

## 2. Materials and Methods

### 2.1. Study Approval

The studies involving mice were authorized by the Italian Ministry of Health, Department of Veterinary Public Health, Nutrition and Food Safety, Directorate General of Animal Health and Veterinary Medicine, as required by the Italian legislation (D.Lgs 26/2014, application of European Directive 2010/63/EU). Protocol number n° 56DC9.92, ministerial authorization n° 126/2024-PR, was used for the in vivo studies in this manuscript.

### 2.2. Mice

Female C57BL/6 J mice, obtained from The Jackson Laboratory (Bar Harbor, ME, USA). Animals were housed in pathogen-free, climate-controlled facilities and provided food and water ad libitum. All efforts were made to minimize the number of animals used and their suffering during the experimental procedure. All animal experiments were supervised by the local Institutional Animal Care Committee (OPBA) of the University of Verona and were conducted according to current European Community rules and approved protocols as mentioned above.

### 2.3. Cell Lines

The RPMI 2650 human nasal carcinoma cell line (ATCC, Manassas, VA, USA) was cultured in Minimum Essential Medium (MEM) supplemented with 100 U/mL penicillin, 100 μg/mL streptomycin, 1% L-glutamine, and 1% no essential amino acids (NEAA) at 37 °C in 5% CO_2_. For the experiments, cells were transferred into a Transwell^®^ membrane (Corning, Grendale, AZ, USA) and cultured in liquid/liquid mode (L/L) for seven days. The RPMI 2650 cell line was then cultured in air/liquid (ALI) mode until reaching appropriated trans-epithelial electrical rResistance (TEER) values (around twenty days), as described in Section 2.5.

The SIM-A9 microglial cell line (ABM Inc., Richmond, BC, Canada) was cultured in Dulbecco’s Modified Eagle Medium/Nutrient Mixture F-12 (DMEM-F12) supplemented with 100 U/mL penicillin and 100 μg/mL streptomycin with 10% FBS, 10% HS at 37 °C in 5% CO_2_.

The SH-SY5Y human neuroblastoma cell line was cultured in DMEM-F12 supplemented with 100 U/mL penicillin and 100 μg/mL streptomycin, 10% FBS and 25 mM of HEPES solution (4-(2-hydroxyethyl)-1-piperazineethanesulfonic acid) at 37 °C with 5% CO_2_.

All reagents for cell cultures were purchased from GIBCO Life Technology (Milan, Italy).

### 2.4. ASCs Culture and ASC-EVs Isolation, Labeling, and Characterization

Murine ASCs were isolated from inguinal adipose tissues of 6–8 weeks old C57Bl/6J mice (Charles River Laboratories, Sant’Angelo Lodigiano, Italy). The stromal-vascular fraction was isolated from the lipoaspirate as previously described [19,29]. ASCs were cultured in DMEM supplemented with 10% FBS, 100 U/mL penicillin, and 100 μg/mL streptomycin at 37 °C in 5% CO_2_. ASC immunophenotypes were assessed by flow cytometry using monoclonal antibodies (mAb) specific for CD106, CD29, CD44, CD80, CD138, and Stem cells antigen 1 (Sca1). The absence of hematopoietic (CD45, CD11c, and CD34) and endothelial markers (CD31) was assessed as previously described [30]. Characterization of ASCs agreed with the MSC standards established by the International Society for Cell Therapy (ISCT) [31]. All mAbs were purchased from Pharmingen/Becton Dickinson (Franklin Lakes, NJ, USA). Isotype-matched antibodies were used as controls.

To prepare fluorescently labelled ASC-EVs (fGFP-EVs), ASC donor cells were infected via lentiviral transduction with a vector expressing farnesylated GFP (#1514 fGFP).

Plasmid #1514 fGFP is a lentiviral vector containing a human PGK promoter driving the expression of the EGFP-CAAX fusion and a CMV enhancer/promoter region involved in viral packaging. The vector includes essential lentiviral elements for genomic integration, such as the 5′ and 3′ LTRs and WPRE (woodchuck hepatitis virus posttranscriptional regulatory element), for improved transcript processing and stability. Cells were sorted by fluorescence and used for fGFP-EV production. Farnesylation of GFP allows GFP to be permanently linked to all cell membranes. Farnesylated GFP (fGFP) is encoded by the fusion of the CAAX-box prenylation motif at the C-terminus of GFP, promoting post-translational farnesylation. Consequently, anchoring the fGFP to the inner leaflet of the plasma membrane allows the fluorescent tagging of membrane-derived extracellular vesicles (EVs), providing stable labeling, as previously reported [32,33]. Increasing concentrations of the lentiviral plasmid during ASC infection, expressed by the multiplicity of infection (MOI) value, were used to control fGFP signal increment in ASC cells.

In some experiments, confluent ASCs were incubated with ultrasmall-iron-oxide-nanoparticles (USPIO, Sigma/Merck, Milan, Italy) at 200 µg Fe/mL for 24 h [34,35]. The efficiency of ASC-EV labeling with USPIO (USPIO-EVs) was previously reported by our group [36].

By using the same protocol for all EV samples, ASC-EVs were isolated from the culture medium at 14–18 passages and characterized as previously described [25]. Briefly, naïve and labeled ASCs were maintained for 48 h in DMEM supplemented with 0.5% exosome-depleted FBS (System Biosciences, Palo Alto, CA, USA). To avoid any FBS contamination, the exosome-depleted FBS supplement was previously ultracentrifugated at 120,000× *g* (Optima Max-E Ultracentrifuge, Beckman Coulter Italy, Milan, Italy) for 18 h, and the collected supernatant was filtered twice through 0.2 µm pore size followed by a third 0.1 µm pore size filtration. ASC supernatants were collected, and small EVs were isolated using a PureExo^®^ Exosome isolation kit (101Bio, Palo Alto, CA, USA) following the manufacturer’s protocol. The isolation and purity of ASC-EVs, USPIO-EVs, and fGFP-EVs were assessed by Western blot. EV pellets were resuspended in RIPA buffer supplemented with protease inhibitors (Roche Diagnostics, Monza, Italy) and incubated on ice for 30 min. The lysates were centrifuged at 14,000× *g* for 15 min at 4 °C. The protein concentration was determined using the enhanced BCA protein assay (Thermo Scientific Pierce, Milan, Italy) in 5 different extractions, according to the manufacturer’s instructions. Absorbance was measured using a spectrophotometer. Then, 30 µg of total protein from fGFP-EVs and 10 µg of protein from parental cell lysates (fGFP-ASCs) were loaded per lane, and the following antibodies were used: polyclonal anti-HSP70 (70 kDa, 1:100 HSP70 (K-20): sc-1060 Santa Cruz Biotechnology, DBA Italia Srl, Milan, Italy), anti-TSG101mAb (45 kDa, 1:1000 Abcam, Cambridge, UK) and polyclonal anti-GM130 Ab (130 KDa, 1:500 Thermo Fisher Scientific, Carlsbad, CA, USA), Alix (96 kDa 1:1000, Abcam, Cambridge, UK). For fGFP-EVs, the presence of GFP protein was evaluated by anti-mouse GFP polyclonal Ab (27 kDa, 1:500, Sigma). After incubation with the appropriate secondary IgG HRP-conjugated antibodies, the membranes were detected with G:BOX F3 GeneSys (Syngene, Cambridge, UK). ASCs lysates were used as a positive control.

Size distribution and concentration of isolated ASC-EVs were measured by Nanoparticle Tracking Analysis (NTA) using a Nanosight NS300 (Malvern Instruments, Worcestershire, UK) equipped with a 488 nm laser and a 500 nm long-pass filter for fluorescence detection. The sample infusion pump was set to a constant flow rate of 20 µL/s. For the measurements, five video recordings with a duration of 1 min were carried out for each sample diluted 1:100 in PBS. In order to achieve a concentration between 20 and 120 particles/frame, the camera level and detection threshold were set in the acquisition and analysis, respectively. The NTA 3.4 software version acquired and analyzed the sample videos. The results are reported as the mean ± SEM of 5 measurements.

### 2.5. In Vitro Model of Nasal Epithelium

RPMI 2650 cells were seeded at 6 × 10^4^ cells/well on a transwell membrane (0.40 mm pore size, Falcon) of a 24 well plate, cultured in ALI mode, and monitored at different time points for trans-epithelial electrical resistance (TEER) by using an EVOM2 volt-ohmmeter (EVOM2, WPI, Sarasota, FL, USA) equipped with electrodes (STX2), until they reached human nasal epithelium values, as described in the literature [37,38]. The characterization of epithelium formation was also monitored by tight junctions’ immunostaining using an anti-occludin antibody (ab31721, 1:50, Abcam, Cambridge, UK). The presence of mucus was detected by Alcian blue staining. The optical images were acquired through an inverted microscope (DM IL LED, Leica, Milan, Italy).

To investigate the potential stimulus exerted by inflammatory conditions on ASC-EV migration across the RPMI 2650 epithelium, 3 × 10^4^ cells/well of SIM-A9 cell line were seeded in the basal compartment of the transwell and treated with lipopolysaccharide (LPS) (2.5 μg/mL) for 24 h.

To verify ASC-EV migration under neuronal oxidative stress and to investigate their potential neuroprotective effect, in the basal transwell compartment of a 24 well plate, 2.5 × 10^4^ SH-SY5Y cells/well were seeded. SH-SY5Y cells were treated with 100 µM hydrogen peroxide (H2O2) for 6 h to induce oxidative stress. SH-SY5Y viability was measured with DAPI (4′,6-diamidino-2-phenylindole) and analyzed by ImageJ (1.53t version) software. The experiments were performed in triplicate for each condition.

In both experimental protocols, 10 µg of fGFP-EVs were added on the top of the transwell to cross the RPMI 2650 cell layer. The quantity of migrated fGFP-EVs through the RPMI 2650 layer was quantified by NTA, as described in Section 2.4. The experiments were performed in triplicate for each condition.

### 2.6. EAE Induction and ASC-EVs Treatment Protocols

Eight-week-old female C57BL/6 J mice were injected subcutaneously with 150 µg MOG35-55 peptide in 200 µL emulsion consisting of equal volumes of PBS and Complete Freund’s Adjuvant (CFA, Difco Laboratories, MI, USA) supplemented with 1 mg of Mycobacterium Tuberculosis strain H37Ra (Difco Laboratories). Mice received 80 ng of Pertussis Toxin (PTx, Alexis Biochemical, San Diego, CA, USA) i.v. at the time of immunization and 48 h later [39]. All mice were weighed and examined daily for clinical scores according to the previously reported scale [20]. Immunized mice were then i.n. treated with (10 μL) containing PBS (control animals) or 5 μg of ASC-EVs accordingly with the following therapeutic regimes: protocol 1 concerning 3 i.n. administrations every 4 days starting from EAE onset and protocol 2 concerning 10 consecutives i.n. administrations starting from EAE onset.

### 2.7. Magnetic Resonance Imaging (MRI)

MR images were acquired using a Bruker Tomograph (Bruker, Karlsruhe, Germany) equipped with a 4.7-T, 33-cm bore horizontal magnet (Oxford Ltd., Oxford, UK). Animals were anesthetized by 1% isofluorane inhalation in a mixture of oxygen and nitrogen and were placed in a prone position over a heated bed. Brain images were acquired with a cross-coil configuration: a volume birdcage coil was used as a transmitter, and a surface helmet coil specific for the mouse brain was used as a receiver. MRI acquisition was performed before i.n. administration in all animals. Then, the animals receive USPIO-EVs at a concentration of 30 µg of proteins (containing 0.2 µg of Fe), resuspended in 20 µL of PBS. USPIO-EVs i.n. injections were performed one day after disease onset for EAE mice and at 50–60 days of life for control animals. The MRI acquisition was performed 3 h after USPIO-EV administration.

In order to detect USPIO-EVs, T2* map images were acquired using a Multi Gradient Echo (MGE) sequence with repetition time (TR) = 2000 ms, echo time (TE) = 3.6 ms, field of view (FOV) = 2 × 1.5 cm^2^, matrix size (MTX) = 160/120, slice thickness = 0.5 mm, n° of slice = number of averages = 9, and total acquisition time = 27 min.

To identify the lesion areas, EAE mice were injected with Magnevist (100 micromol/kg), and T1-weighted RARE images were acquired with the following parameters: TR = 926 ms; TE-eff = 19.1 ms; FOV = 2.0 × 1.5 cm^2^; MTX = 192 × 144; slice thickness = 0.5 mm; n° of slice = 25; RARE factor = 3; number of averages = 12. T1-weighted images were acquired before and after the Gd-based contrast agent injection.

The presence of USPIO-EVs was also verified by histological analysis on frozen brain sections of EAE-induced mice. Briefly, animals were deeply anesthetized and transcardially perfused with 4% paraformaldehyde. Brains were dissected, and 2 h post-fixation with 4% paraformaldehyde, they were soaked in 30% sucrose, included in OCT, and serially cut at 10 µm thick with a cryostat apparatus. To evaluate EV localization, tissue sections were incubated with a Prussian Blue solution (5% hydrochloric acid and 5% potassium ferrocyanide) for 40 min and counterstained with nuclear fast red for 10 min. Sections were examined with a Zeiss Axiophot microscope equipped with an Axiocam HCR camera and Axiovision software (version 4.3). Images were acquired from representative sections of 10 µm thick of mice/collected every 50 µm.

### 2.8. Neuropathology

At the experimental endpoint for both therapeutic protocols, mice were deeply anesthetized and transcardially perfused for spinal cord explant and freezing. The lumbar spinal cord was serially cut at 15 µm thick with a cryostat apparatus for immunohistochemistry and immunofluorescent staining.

Tissue sections were pre-incubated for 10 min in 3% H_2_O_2_ to quench endogenous peroxidase and then incubated for 1 h in 20% NGS and 1% BSA in PBS. Slides were incubated overnight at 4 °C with the following primary antibodies diluted in 1% NGS in PBS: anti-CD3 (1:500, MCA500; Bio-Rad, Hercules, CA, USA), anti-mouse GFAP (1:500, Z0334 Dako, Santa Clara, CA, USA), and anti-Iba1 (1:500 019–19,741 Wako). Tissue sections were then incubated for 1 h in biotinylated goat anti-rabbit IgG (1:100, Vector Laboratories, Newark, CA, USA). The avidin-biotin-peroxidase kit (ABC kit; Vector) and Novared kit (Vector Laboratories) were used as signal revelation systems. Slides were dehydrated through increasing grades of ethanol, cleared in xylene, and mounted with a coverslip using Entellan (Merck, Darmstadt, Germany). For the analysis, CD3, GFAP, or Iba1-positive cells were counted every 100 µm; images were acquired using a LEICA microscope at a magnification of 20× and blindly counted using ImageJ software (1.53t version, U. S. NIH, Bethesda, MA, USA).

Histological assessment of spinal cord demyelination (Woelcke staining) in lumbosacral segments was blindly performed, calculating affected areas in at least three sets (100 μm apart) with ImageJ software.

For immunofluorescence staining, the lumbar spinal cord sections were incubated in a blocking solution with 5% NGS and 1% BSA in PBS for 1 h at RT. The sections were incubated overnight at 4 °C with the following primary antibodies diluted in 1% NGS in PBS: anti-SMI32 (1:1000, BioLegend, San Diego, CA, USA). Alexa Fluor anti-mouse 488 (1:800, Invitrogen/Thermo Fisher Scientific, Carlsbad, CA, USA) was used. For the analysis of neurodegeneration, section images of SMI-32-positive cells were converted into black-and-white images. Then, the density of immunopositive profiles in the total area was quantified using Image J and expressed as a percentage.

### 2.9. Multiplex Protein Analysis

At the experimental endpoints, mice were intracardially perfused with PBS. Supernatants from homogenates (4 μL diluted 1:15) of spinal cord and brain isolated from EAE mice treated with ASC-EVs or PBS were used for the assay using Mouse Cytokine 23-Plex (#M60009RDPD, Bio-Rad) magnetic bead panels in a Bio-Plex X200 Luminex instrument equipped with a magnetic workstation (Biorad, Hercules, CA, USA), following the manufacturer’s instructions. Bio-Plex software (version 6.0) automatically calculated cytokines and chemokine concentrations using a standard curve derived from recombinant standards. The level of each protein detected during the analysis was normalized to the total protein concentration of each brain or spinal cord sample (measured by Bradford protocol). All samples were run in duplicate in the same experiment.

### 2.10. Statistical Analysis

All data were expressed as the mean ± standard error (SEM). Statistical analysis was performed using a two-tailed Student’s *t*-test or one-way ANOVA with Tukey’s or Bonferroni’s correction. For multiplex analysis and EAE parameters, the Mann–Whitney test was used. For the EAE clinical score, a two-tailed Kolmogorov–Smirnov test was applied. All analyses were performed with GraphPad Prism 8. A *p*-value < 0.05 was considered statistically significant.

## 3. Results

### 3.1. Inflammation Drives ASC-EV Diffusion Through the Nasal Epithelium

**Figure 1:**
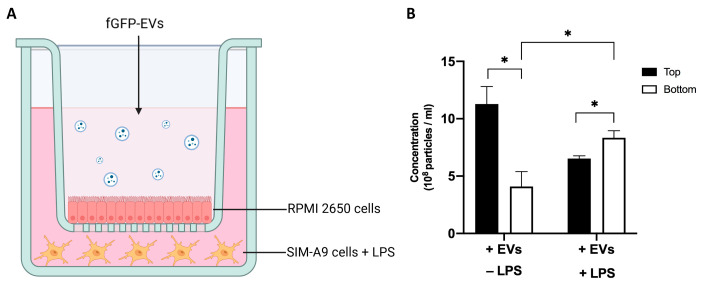
Ability of fGFP-EVs to migrate across epithelia attracted by inflammatory signals. (**A**) Schematic representation of in vitro transwell system. (**B**) NTA quantification in terms of EV concentrations (particles/mL) in the supernatant of the upper and lower compartment of the transwell after 6 h of fGFP-EVs treatment. SIM-A9 cells +/− LPS were plated in the bottom of the transwell. Data are represented as the mean of cell viability ± SEM of three different experiments. Ordinary one-way ANOVA with Tukey’s correction was performed between all experimental conditions for each treatment (* *p* < 0.05).

It is well established that chemotactic signals released during inflammation increase stem cell recruitment to sites of inflammation [40,41]. In this regard, we evaluated if an inflamed microenvironment could attract ASC-EVs, promoting their transit through epithelia. For this purpose, we used an in vitro model of nasal epithelium in a transwell system where RPMI 2650 cells were cultured on the membrane, growing in ALI mode. Before the experiment, an extensive characterization of the epithelium was performed, and consistent features for cellular epithelium formation and integrity were reported at around 21 days (Supplementary Figure S1). This timing was used to perform all the in vitro experiments. In the bottom chamber of the transwell, LPS-activated SIM-A9 cells were seeded (Figure 1A). fGFP-EVs were generated transfecting ASCs with a construct encoding for a farnesylated GFP and seeded on a RPMI 2650 layer (Supplementary Figure S2). After 6 h, fGFP-EV migration below the epithelium was evaluated by NTA using an additional 500 nm longpass filter for specific fluorescent EV detection. A significant increase (*p* = 0.035) in fGFP-EVs was detected in the supernatant of the lower chamber in LPS-treated SIM-A9 cells compared to the supernatant of unstimulated control SIM-A9 (Figure 1B). These data suggest that inflammatory signals recall and attract ASC-EVs, facilitating their migration through the epithelia.

**Figure 2:**
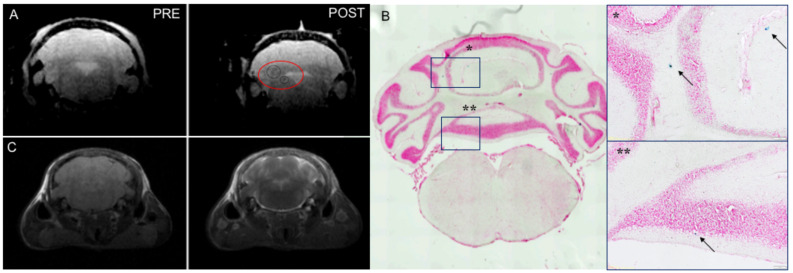
Detection of USPIO-EVs in inflamed sites of EAE mouse brain after intranasal injection (**A**) In vivo MRI images (T2* map) acquired pre- and post-intranasal injection of USPIO-EVs in EAE mice. The iron nanoparticles that indicate the presence of small extracellular vesicles, were detected at 3 h post injection (circles). (**B**) The Prussian blue staining confirmed the presence of labelled vesicles in the region detected by MRI. Scale bar = 100 μm. and indicate magnified views of the corresponding brain areas. Black arrows point to Prussian blue–positive signals. (**C**) T1-weighted images of Gadolinium-based contrast agent-identified inflamed lesion area.

To confirm these observations in vivo, USPIO-EVs were i.n. administered in healthy control mice and EAE mice one day after disease onset and visualized by MRI 3 h post-treatment. The signal was detected in the brain during EAE, preferentially located in positive for Gd-based contrast agent-inflamed areas (Figure 2A,C). No specific signals were observed in healthy mice, as demonstrated by our previous publication [34]. Congruently, histological analysis by Prussian Blue staining confirmed imaging data showing the presence of positivity in the same areas revealed by MRI (Figure 2B).

### 3.2. ASC-EVs Exert Neuroprotective Effects on Injured Neurons Ater Their Passage Through the Nasal Epithelium

**Figure 3:**
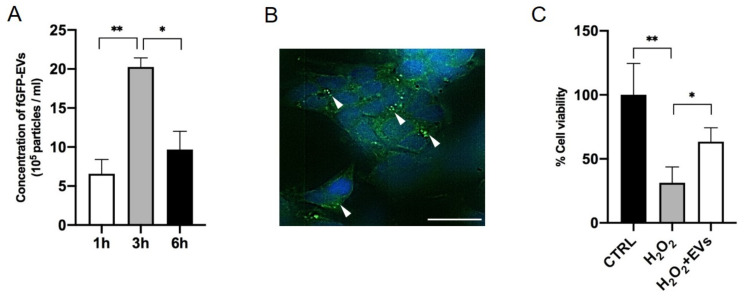
Neuroprotective effect of fGFP-EVs on injured SH-SY5Y cells after their passage through the nasal epithelium (**A**) NTA quantification of fGFP-EVs (particles/mL) in the supernatant of the lower compartment of the transwell at different time points. (**B**) fGFP-EV detection in injured SH-SY5Y cells by confocal microscopy. Scale bar = 10 μm. White arrows point to fGFP-EVs signals. (**C**) Cell viability of SH-SY5Y injured with 100 µM H_2_O_2_, after 6 h of fGFP-EV treatment. Data are represented as the mean of cell viability ± SEM of three different experiments. Ordinary one-way ANOVA with Bonferroni’s correction was performed between all the experimental conditions for each treatment (* *p* < 0.05 and ** *p* < 0.001).

To evaluate the capability of fGFP-EV migration across the epithelium reaching injured sites, we set up a transwell system similar to the one previously described, in which the bottom of the chamber was seeded with oxidative-stressed SH-SY5Y cells. After fGFP-EV seeding on the RPMI 2650 layer, NTA analysis of the below medium was performed at different timings. A significant increase of fGFP-EVs detected from 1 to 3 h indicated the ability of fGFP-EVs to cross the epithelium in a time-dependent manner. Although after 6 h of treatment, the amount of fGFP-EVs was reduced in the supernatant (Figure 3A), at this time point, we found the presence of green fluorescent spots inside SH-SY5Y cells, suggesting that EVs were internalized by the damaged cells (Figure 3B). Of note, fGFP-EVs endocytosis correlated with increased neuronal rescue. The viability of SH-SY5Y cells was, indeed, significantly improved in the presence of fGFP-EVs (*p* = 0.0464) if compared to oxidative stress-induced SH-SY5Y cells cultured in the absence of fGFP-EVs (Figure 3C). These data indicate that cellular stress signals could induce fGFP-EV migration through the epithelial layer and their subsequent SH-SY5Y cell internalization with a neuromodulatory outcome.

### 3.3. Intranasal Administration of ASC-EVs Reduces EAE Severity

**Figure 4:**
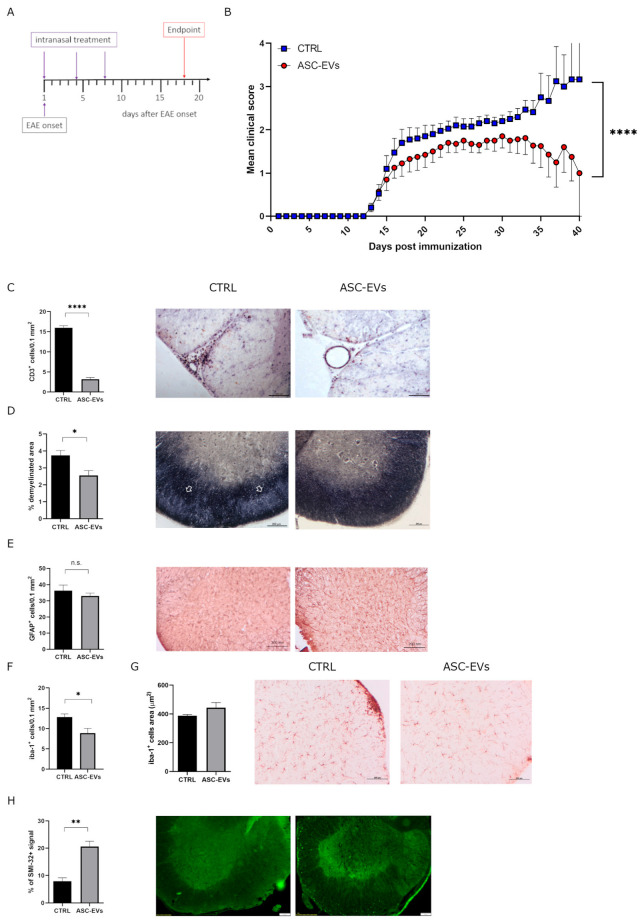
Clinical and neuropathological assessment of ASC-EV i.n. administration in EAE mice (**A**) Schematic representation of the experimental protocol. (**B**) Clinical score of MOG35-55-induced EAE mice treated i.n. with PBS (vehicle) or with ASC-EVs. Mice were, one at a time, i.n. treated at the EAE onset and individually sacrificed once the experimental endpoint was achieved. Data are represented as the mean ± SEM of 20 mice/condition. Two-tailed Kolmogorov–Smirnov test was used (**** *p* < 0.0001). Quantification and representative images of histological analysis for (**C**) CD3+ cells, (**D**) demyelination (Woelcke staining), (**E**) GFAP+ and (**F**,**G**) Iba-1+ cells (Scale bar 200 µm) and (**H**) SMI-32 (Scale bar 100 μm) 10 days after the last ASC-EV administration. Data are represented as the mean ± SEM of 5 mice/condition (* *p* < 0.05, ** *p* < 0.001, **** *p* < 0.0001).

To compare the therapeutics of ASC-EVs i.n. administrated with that of i.v. administration as previously described [20], we treated EAE-induced mice 3 times every 4 days starting from disease onset (Figure 4A). Due to the substantial temporal window for EAE onset (Table 1), we decided to treat mice individually at the first appearance of clinical symptoms. EAE mice treated with ASC-EVs showed a significant amelioration in clinical severity compared to PBS-treated control mice (*p* < 0.0001) (Figure 4B). The therapeutic effect of ASC-EVs particularly impacted disease severity at the end of the treatment, as shown by similar means of the cumulative score and maximum clinical score but significantly different mean clinical score at the experimental endpoint compared to the control group (*p* = 0.0325) (Table 1). Moreover, 25% of ASC-EV-treated mice reached complete clinical remission (Table 1).

Ten days after the last ASC-EV administration, mice were euthanized, and in some of them, the spinal cord was explanted for neuropathology assessment. From a histopathological point of view, disease amelioration in ASC-EV-treated mice was associated with a drastic reduction in T lymphocyte infiltration at the lumbar spinal cord level (*p* = 0.0020) (Figure 4C) associated with a substantial demyelination reduction (*p* = 0.025) (Figure 4D) if compared to control EAE-induced mice. Besides, microglial cells decreased following the treatment with ASC-EVs (*p* = 0.0146), which did not significantly affect their activation status (Figure 4F,G). No remarkable changes in the number of astrocytes were reported (Figure 4E). Interestingly, motoneuron neurofilaments from the ventral horns of the spinal cord’s gray matter were remarkably preserved compared to control mice (*p* = 0.0077), suggesting the potential neuroprotective role of ASC-EVs in preventing axonal damage (Figure 4H).

### 3.4. Intranasal Injection of ASC-EVs Induces Immunomodulation in CNS During EAE

**Figure 5:**
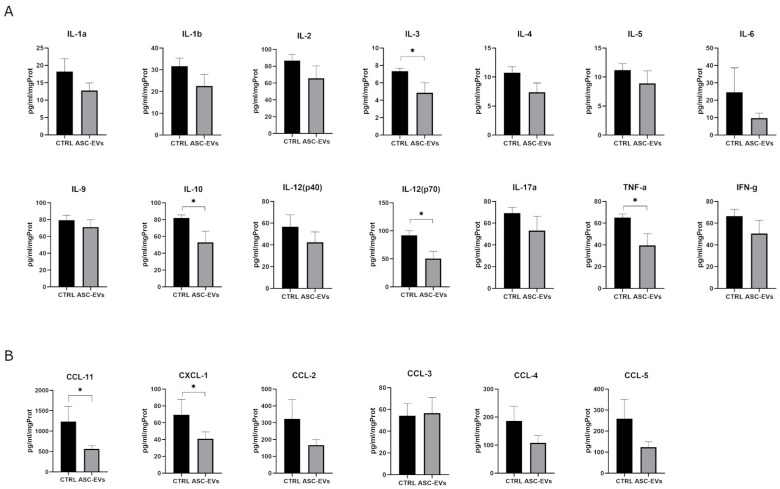
Cytokine and chemokine detection. Multiplex analysis of (**A**) cytokines and (**B**) chemokines detected in spinal cord homogenates of EAE mice treated with ASC-EVs and PBS-injected controls. The protein concentration of the molecules is expressed as pg/mL/mg of the total protein content. Data are represented as the mean ± SEM of one representative experiment from a series of two similar results for *n* = 5 mice/condition by Mann–Whitney test (* *p* < 0.05).

To investigate processes by which ASC-EVs could affect the EAE outcome, we investigated the production of pro- and anti-inflammatory cytokines and chemokines in the spinal cord and brain homogenates of EAE mice sacrificed 10 days after the administration protocol. If compared to the spinal cord of control mice, ASC-EV treatment induced lower degrees of pro-inflammatory cytokines, with peculiar significant decreased TNF-a (*p* = 0.0286) and IL-12 (p70) (*p* = 0.0286) production in the spinal cord (Figure 5A). However, ASC-EV treatment had also reduced the production of several anti-inflammatory cytokines, such as IL-4, and IL-5, including a significantly decreased IL-10 delivery (*p* = 0.0317), suggesting a more generalized immunomodulatory effect exerted by ASC-EVs in the spinal cord during EAE (Figure 5A). To note, less evident immunomodulation was detected in brain homogenates, perhaps because of the lower inflammatory state if compared to the spinal cord (Supplementary Figure S3A), which is considered the primary site of inflammation in the chronic EAE mouse model. Interestingly, IL-3, which was particularly affected after ASC-EV administration (*p* = 0.0317), has been recently involved in glial–peripheral immune interplay in CNS plaques and correlated to exacerbated MS and EAE pathology (Figure 5A) [42].

On the other hand, cytokines were also modulated by ASC-EVs. Spinal cord and brain homogenates obtained from ASC-EV-treated mice displayed a tendency in CCL and CXCL chemokines to decrease with significantly reduced levels of CCL-11 (*p* = 0.0286), and CXCL-1 (*p* = 0.049) in spinal cord as in brain (*p* = 0.0317; *p* = 0.0317 for CCL-11 and CXCL-1 respectively) (Figure 5B and Supplementary Figure S3B). These data partially explain the robust reduction in T cell infiltration (Figure 4C).

### 3.5. Daily Intranasal Administration of ASC-EVs Reduces Clinical Severity and Neuropathological Features During EAE

**Figure 6:**
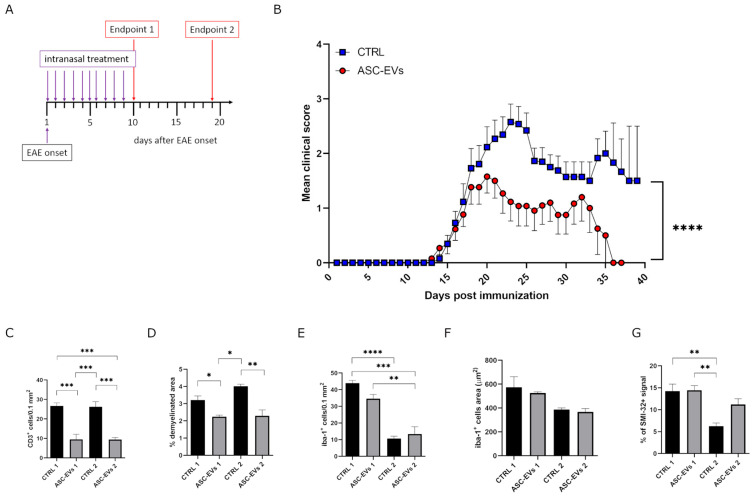
Clinical and neuropathological assessment of daily ASC-EV i.n. administration in EAE mice. (**A**) Schematic representation of experimental protocol. (**B**) Clinical score of MOG35-55 induced EAE mice treated i.n. with PBS (vehicle) or with ASC-EVs. Mice were, one at a time, i.n. treated at EAE onset and individually sacrificed once the experimental endpoints were achieved. Data are represented as the mean ± SEM of 13 mice/condition. Two-tailed Kolmogorov–Smirnov test was used (**** *p* = 0.0001). Quantification and histological analysis for (**C**) CD3+ cells, (**D**) demyelination (Woelcke staining), (**E**,**F**) Iba-1+ cells and (**G**) SMI-32 one day after the last administration (endpoint 1), or 10 days later (endpoint 2). Data are represented as the mean ± SEM of 3 mice/condition (* *p* < 0.05, ** *p* < 0.001, *** *p* < 0.0005, **** *p* < 0.0001). Representative images are reported in Supplementary Figure S5.

In light of promising results obtained using the intranasal delivery approach combined with our validated treatment regimen [20], we decided to move further, testing a new therapeutic protocol involving 10 consecutive days of treatment starting at the EAE onset (Figure 6A). We performed a pilot experiment showing a significant improvement in the therapeutic effect exerted by ASC-EVs on clinical manifestation severity throughout the disease course (*p* = 0.0001) (Figure 6B). These data were also supported by clinical feature estimation, showing a significant mean reduction in maximum clinical score and cumulative disease index in ASC-EV-treated mice compared to the control group (*p* = 0.0262 and *p* = 0.0053, respectively) (Table 2). Some mice were euthanized one day after the last administration (endpoint 1; Supplementary Figure S4A), while the remaining 10 days later (endpoint 2; Supplementary Figure S4B), and spinal cords were obtained for histology assessment.

The number of infiltrating T cells in association with demyelination degree was already found to be widely reduced one day after the end of treatment (*p* = 0.0004 and *p* = 0.0477 CTRL 1 vs. ASC-EVs 1, respectively). This reduction was maintained for at least another 10 days (*p* = 0.0005 CTRL 2 vs. ASC-EVs 2 for T cell infiltration and *p* = 0.0081 CTRL 2 vs. ASC-EVs 2, respectively) (Figure 6C,D).

Microglia were reduced immediately after ASC-EV administration but in contrast to the previous therapeutic protocol, not at the later time point (Figure 6E,F). Notably, the considerable microglial cell decline occurring between endpoints 1 and 2 (*p* < 0.0001 in control mice and *p* = 0.0013 in treated mice) was already correlated to clinical progression in chronic EAE (Figure 6E) [43]. As expected, no effect on neurofilament detection was found at the early time point from daily treatment. However, the partial later recovery from axonal damage suggests a protective contribution by ASC-EV handling on the neurodegenerative process during EAE progression (Figure 6G).

## 4. Discussion

Neurodegenerative disorders, such as multiple sclerosis (MS), Alzheimer’s disease (AD), and Parkinson’s disease (PD), denote a heterogeneous group of diseases in which inflammation is increasingly emerging as a critical pathogenetic feature [44]. In these contests, the anti-inflammatory and neuroprotective properties of MSC-EVs represent a promising alternative strategy of cell-free treatment for neurological healing [22,45].

Several works indicate that i.v. injection of MSC-EVs in different animal models of stroke may lead to enhanced white matter integrity by neuronal remodeling, synaptic plasticity, neurogenesis, neoangiogenesis, and oligodendrogenesis [46,47,48]. However, another study has associated neuroprotection after stroke with reduced leukocyte CNS infiltration [49].

MCS-EV i.v. administration also improved cognitive impairment in different AD-mouse models by inducing hippocampal neurogenesis or enhancing neuron resistance from oxidative stress damage [50,51]. Mainly, intracerebral injected MSC-EVs induce Aβ proteolysis and its microglia phagocytosis, accordingly reducing Aβ deposition and plaque load, one of the pathological disease hallmarks [52].

Moreover, in transgenic rat models of PD, MSC-EVs induce extracellular α-synuclein degradation, preserving dopaminergic neuron viability with significant motor deficit recovery [53,54,55].

MS is a chronic inflammatory neurological disorder with an autoimmune origin characterized by neuropathological features, such as perivascular infiltration, microgliosis, and astrogliosis, leading to progressive myelin degradation and variable degrees of axonal degeneration [56], whose most widely used animal model is the experimental autoimmune encephalomyelitis (EAE) [57]. In EAE-induced mice, the immunomodulation of MSC-EV treatment mitigates the disease clinical score [20,58,59]. On the one hand, MSC-EVs reduce T cell proliferation, proinflammatory cytokine release, and migration into the CNS; on the other hand, they enhance anti-inflammatory cytokine production and increase T regulatory CD4+CD25+Foxp3+ frequency [20,58,59,60]. Although in an EAE model of chronic progressive neurodegeneration, where demyelination is induced by infecting SJL mice with Theiler’s murine encephalomyelitis virus (TMEV), MSC-EV handling improves motor activity, reducing gliosis and astrocyte activation, raising neuron remyelination [61]. We previously demonstrated that in chronic EAE-induced mice, ASC-EVs efficiently ameliorate disease severity when administered preventive i.v. injected at the preclinical phase by reducing peripheral autoreactive T cell activation and migration into the CNS. Unlike what was observed by Jafarinia and colleagues [58], ASC-EVs therapeutically administered starting from the disease onset were useless for EAE modulation, suggesting that the peripheral immunomodulation exerted by ACS-EVs was enough to ameliorate EAE in the preventive protocol of administration but not sufficient for a therapeutic treatment [20]. Interestingly, we also indicated that USPIO-labelled ACS-EVs, after i.v. injection rapidly accumulates in the lymph nodes of EAE-induced mice, where they target resident antigen-presenting cells, but not in the CNS [25]. These data contrast with our previous study we conducted on whole mesenchymal stem cells where both preventive and therapeutic protocols of ASCs, administrated i.v., were effective in ameliorating EAE, reaching both lymph nodes and CNS [29], as confirmed by a subsequent study carried out by Shalaby et al. [62]. Notably, ASCs could actively migrate into the CNS in a VLA-4-dependent manner, improving oligodendrocyte survival [29], suggesting that the integrin machinery could poorly support parental EV migration to the CNS. Conversely, pharmacokinetic studies on exogenously administered EVs have elucidated that their biodistribution depends on the route of administration [63,64]. Also, it was observed that, after i.v. injection in mice models, EVs preferentially accumulate in the liver, gastrointestinal tract, lungs, spleen, and lymph nodes, and this commission relies on the cells of origin [20,25,63,65]. Independent of the pathological context in which they were investigated, it was widely demonstrated that resident macrophages mainly take up EVs [25,63,66,67,68,69,70]. Although EVs could bypass an in vitro model of BBB [71], these data indicate that resident macrophages, playing a role in sequestering exogenous EVs from the circulation after their systemic administration, could prevent an efficient diffusion into the CNS. In light of these data, intranasal administration represents a promising strategy to improve EV delivery in the CNS for neurological disorders [72,73,74,75,76].

Our in vitro data exploited a commonly used model for drug transportation studies, the RPMI 2650 cells [77,78], to verify and monitor ASC-EVs’ passage through the epithelium as initially occurs after intranasal administration. We observed an increase in the amount of ASC-EVs able to cross the epithelium in the experimental conditions where inflammatory stimuli raised recall. This evidence is in line with several studies where inflammatory processes have been shown to trigger the entry of extracellular vesicles into the brain [79], promoting their accumulation in damaged/inflamed areas of the brain [75]. Similarly, we observed that i.n. injection of ASC-EVs conjugated with ultra-small iron oxide nanoparticles (USPIO-EVs) in EAE mice enabled the detection of the MRI signal preferentially located in inflamed and injured brain areas. This method allows non-invasive monitoring of ASC-EVs, overcoming several limitations of extracellular vesicle imaging that could allow their tracking and quantification in specific brain regions [34,36]. Although inflammation can trigger ASC-EV recruitment, neurons represent one of their preferential targets [75]. In our experimental setting, ASC-EVs could cross the epithelium, growing quantities within 3 h of their administration and subsequently being absorbed by damaged neurons; therefore, they were poorly detectable in the culture medium at later times. Following their neuronal uptake, ASC-EVs significantly promote their rescue from oxidative damage, as previously demonstrated in neuroblastoma cells [21] and motor neuron cell lines transfected with pathological ALS mutations [19].

In order to improve EV diffusion into the CNS of EAE mice, boosting their neuroprotective functions, we intranasally treat EAE-induced mice with the same therapeutic protocol, in which EVs failed to ameliorate EAE when i.v. injected [20]. We observed long-term disease amelioration concerning clinical disability and neuropathological features. Reduction in CNS T cell infiltration and demyelination in concomitance with increased detection of motoneurofilaments suggests an immunomodulatory and neuroprotective function exerted by intranasally administrated ASC-EVs. Interestingly, in the spinal cord, after ASC-EV i.n. administration, we observed a reduction of cytokines and chemokines involved in EAE and MS pathogenesis and progression [80], suggesting a mitigation of the occurring inflammatory processes. Moreover, it was also demonstrated that cytokines, such as Il-1b and TNF-a, could act directly as neuromodulators, inducing synaptopathy in EAE [80]. In this context, IL-9, which plays a protective role in synaptic damage by reducing synaptotoxic TNF-a release, was unaffected by ASC-EV administration in the spinal cord and brain (Figure 5A and Supplemental Figure S3A) [81]. We also observed reduced chemokine levels after ASC-EV treatment in both the spinal cord and brain; these data align with a decreased infiltrating pattern. However, recent data argue that chemokines are directly involved in neurotransmission signaling and synaptic plasticity, as neurons could express their counter ligands [82,83]. Nevertheless, their physiologic role could degenerate into neurotoxic effects during chronic neuroinflammation, as demonstrated in a murine model of Alzheimer’s disease for CCL2/CCR2 and CXCL1/CXCR2 [84,85]. Moreover, it was in vitro demonstrated that CCL11, one of the significantly decreased chemokines in the CNS after i.n. ASC-EV treatment could directly suppress the proliferation of neuronal progenitor cells [86]. Besides, CCL11 enhances neuronal excitotoxicity by microglia ROS production and, directly, synaptic toxicity and tau phosphorylation in adult differentiated CNS cells [85,87,88].

All these data suggest that ASC-EVs exert immunomodulatory outcomes that could also impact neuroprotective processes during EAE.

Identifying the dosage and frequency of EV-based treatments to achieve and maximize the therapeutic effect is extraordinarily complex, and the results are highly variable, slowing down the translation process towards the clinic [89,90]. In this context, the intranasal administration represents a flexible strategy, which simplifies the delivery procedure. Among the many advantages, the intranasal route of administration allows for repeated administrations to improve the therapeutic effects of EVs without reporting significant side effects. In this regard, we evaluated a daily injection protocol for ASC-EV administration in a pilot experiment to identify the correct therapeutic intervention window. Our data suggest that daily intranasal administration is well tolerated in mice. Although there is a less significant long-term effect on microglial cells and neurofilament preservation, probably due to the low number of animals recruited for the experiments, we observed enhanced therapeutic effects of ASC-EVs, as suggested by a significant decline in disease parameters, such as maximum clinical score and cumulative disease index, in ASC-EV treated mice compared to the control group.

A very rapid brain spreading of MSC-EVs was observed in healthy mice after intranasal delivery. EVs were found distributed far from the olfactory bulb, preferentially accumulating in subcortical residence cells [91]. Interestingly, Perets and colleagues highlighted how, after their intranasal administration in different brain pathologies, including stroke, AD, PD, and autism, MSC-EVs were attracted by neuroinflammatory signals, leading to a long-term disease pattern distribution [75]. The use of the i.n. route is suitable for progressive or recurrent diseases requiring repeated treatments to obtain long-term benefits. Daily insulin administration by the intranasal route has already been adopted in AD patients [92]. Currently, an ongoing phase I/II clinical trial by Wang and colleagues [93] evaluates the safety and efficacy of MSC-EVs in patients with mild to moderate dementia by repeated intranasal administration of MSC-EVs. The possibility of adopting therapeutic regimens of repeated administrations with the intranasal modality translates into an improvement in patient tolerance without the potential side effects reported by more invasive approaches. Moreover, it could promote the development of devices for self-administration or simple formulations that can be used autonomously even by patients affected by cognitive and functional problems [94].

Although EVs represent the paracrine effectors through which MSCs exert their immunomodulatory and regenerative properties, we may deduce that they can maintain the same capabilities as the cells from which they originate [95,96,97,98]. Accumulating studies corroborated how EVs could sustain the same beneficial effect exerted by their parental MSCs [22,45,58,99], avoiding all the side effects of live cell therapies [100,101]. From a physiological point of view, EVs exhibit a higher safety profile, disclosing lower tumorigenicity and immunogenicity and higher biocompatibility and tissue spreading capacity. Otherwise, from a manufacturing perspective, EVs maintain their bioactivity during culture expansion and frozen storage. Moreover, they might be persistently secreted from immortalized MSCs or MSCs derived from human-induced pluripotent stem cells (iPSCs) for better standard reproducibility and unlimited production [17,102].

In conclusion, our data show that intranasal treatment of ASC-EVs could represent an excellent cell-free therapeutic approach with a potential successful short-term clinical translation in MS, as in other chronic neurodegenerative disorders in which inflammation plays a critical role. Therefore, safe and standard MSC-EV processing challenges must still be addressed to render the research prompt to the clinic [103].

## Figures and Tables

**Table 1:** Clinical features of EAE mice treated with 3 i.n. administration every 4 days starting from disease onset. Experimental endpoint: 10 days after the last administration. Data are from 2 independent experiments and represented as the mean ± SEM. * *p* < 0.05 by Mann–Whitney t test. n.s. = not significant.

Disease Parameters	CTLR	ASC-EV Treatment	*p* Value
Number of animals	20	20	
Remission incidence	0/20	5/20	
Disease onset (d.p.i) (range)	16.0 ± 0.6508 (13–22)	16.3 ± 0.7000 (13–22)	n.s.
Maximum clinical score (range)	2.9 ± 0.2606 (1.5–4.5)	2.6 ± 0.2529 (1–5)	n.s.
Cumulative disease index (range)	42.45 ± 3.047 (25.5–75)	32.55 ± 4.018 (2.5–67)	n.s.
Mean clinical score endpoint (range)	2.37 ± 0.1983 (1–4.5)	0.3 ± 0.2291 (0–3)	* *p* = 0.0325

**Table 2:** Clinical features of EAE mice treated with 10 consecutive i.n. administrations starting from disease onset. Experimental endpoint 1: 1 day after the last administration; experimental endpoint 2: 10 days after the last administration. Data are from 1 experiment and values are represented as the mean ± SEM. * *p* < 0.05; ** *p* < 0.005 by Mann–Whitney t test. n.s. = not significant.

Disease Parameters	CTLR	ASC-EV Treatment	*p* Value
Number of animals	13	13	
Remission incidence	1/13	6/13	
Disease onset (d.p.i) (range)	16.9 ± 0.5934 (14–21)	16.8 ± 0.6489 (13–20)	n.s.
Maximum clinical score (range)	2.85 ± 0.2852 (1.5–4.5)	2.08 ± 0.2392 (1–4)	* *p* = 0.0262
Cumulative disease index (range)	32.38 ± 3.962 * (9–52.5)	18.23 ± 4.694 * (2.5–65)	** *p* = 0.0053
Number of animals endpoint 1	6	8	
Mean clinical score endpoint 1 (range)	2.42 ± 0.4362 * (1.5–4.5)	1 ± 0.3536 * (0–2.5)	* *p* = 0.0183
Number of animals endpoint 2	7	5	
Mean clinical score endpoint 2 (range)	1.57 ± 0.3689 (0–2.5)	1.1 ± 0.6782 (0–3)	n.s.

## Data Availability

The data presented in this study are available on request from the corresponding author.

## Supplementary Materials

The following supporting information can be downloaded at: https://www.mdpi.com/article/10.3390/cells14151172/s1, Figure S1: Characterization of the in vitro model of the epithelium; Figure S2: Characterization of fGFP-ASC and fGFP-EVs; Figure S3: Cytokine and chemokine detection; Figure S4: Clinical features of daily ASC-EV i.n. administration in EAE mice; Figure S5: Lumbar spinal cord histology of daily ASC-EV i.n. administration in EAE mice.

## Abbreviations

The following abbreviations are used in this manuscript:

ASCs	Adipose stem cells
MS	Multiple sclerosis
ASC-EVs	Adipose-derived extracellular vesicles
EVs	Extracellular vesicles
i.v.	Intravenous
i.n.	Intranasal
EAE	Experimental autoimmune encephalomyelitis
CNS	Central nervous system
Gd	Gadolinium
USPIO	Ultrasmall superparamagnetic iron oxide
BBB	Blood–brain barrier
fGFP	Farnesylated green fluorescent protein
NTA	Nanoparticle tracking analysis
TEER	Trans-epithelial electrical resistance
MRI	Magnetic resonance imaging
