# Supplementary material for: Intranasal Administration of Extracellular Vesicles Derived from Adipose Mesenchymal Stem Cells Has Therapeutic Effect in Experimental Autoimmune Encephalomyelitis
Source: Cells. 2025 Jul 30;14(15):1172. doi: 10.3390/cells14151172 (PMC12345849; doi:10.3390/cells14151172)
Supplement: Supplementary file 1 [file cells-14-01172-s001.zip › cells-3750540-supplementary.pdf]

cells

MDPI

Article

Intranasal Administration of Extracellular Vesicles Derived from Adipose Mesenchymal Stem Cells Has Therapeutic Effect in Experimental Autoimmune Encephalomyelitis

Barbara Rossi [1,*,†], Federica Virla [2,†], Gabriele Angelini [1], Ilaria Scambi [2], Alessandro Bani [1], Giulia Marostica [3], Mauro Caprioli [2], Daniela Anni [2], Roberto Furlan [3], Pasquina Marzola [4], Raffaella Mariotti [2], Gabriela Constantin [1], Bruno Bonetti [5] and Ermanna Turano [2,*]

[1] Division of General Pathology, Department of Medicine, University of Verona, 37129 Verona, Italy; gabriele.angelini@univr.it (G.A.); alessandro.bani@univr.it (A.B.); gabriela.constantin@univr.it (G.C.)
[2] Department of Neuroscience, Biomedicine and Movement Sciences, University of Verona, 37129 Verona, Italy; federica.virla@univr.it (F.V.); ilaria.scambi@univr.it (I.S.); mauro.caprioli@univr.it (M.C.); daniela.anni@univr.it (D.A.); raffaella.mariotti@univr.it (R.M.)
[3] Clinical Neuroimmunology unit, Institute of Experimental Neurology, San Raffaele Scientific Institute, 20132 Milan, Italy; giulia.marostica@hotmail.it (G.M.); furlan.roberto@hsr.it (R.F.)
[4] Department of Engineering for Innovation Medicine, University of Verona, 37129 Verona, Italy; pasquina.marzola@univr.it
[5] Neurology Unit, Azienda Ospedaliera Universitaria Integrata Verona, 37126 Verona, Italy; bruno.bonetti@aovr.veneto.it
* Correspondence: barbara.rossi@univr.it (B.R.); ermanna.turano@univr.it (E.T.)
† These authors contributed equally to this work.

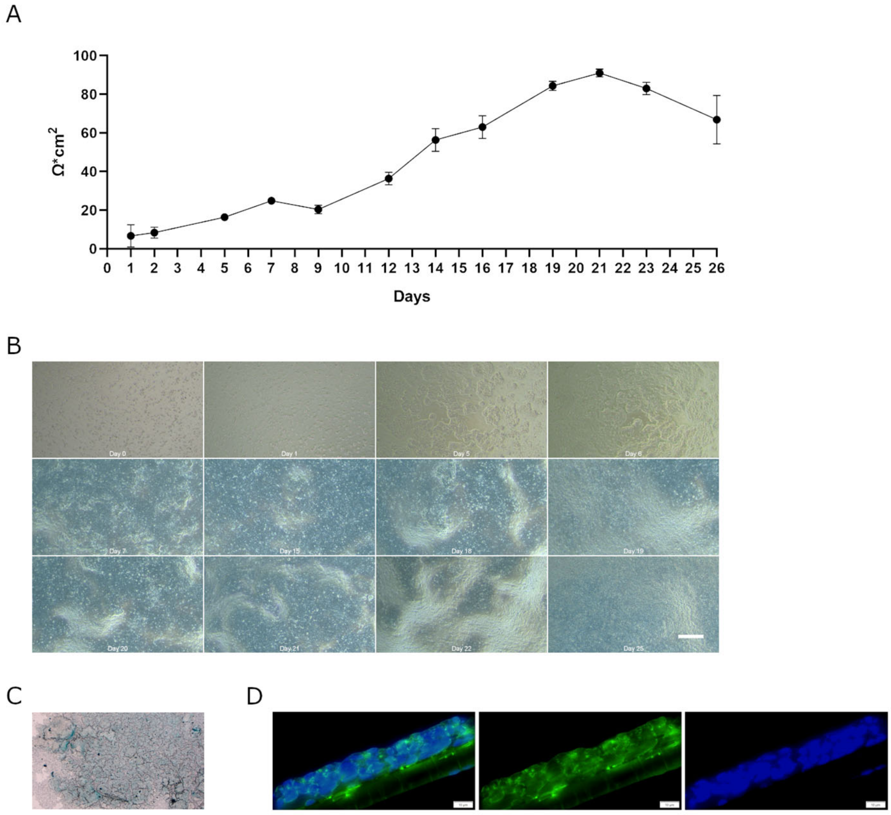

Figure S1. Characterization of the *in vitro* model of epithelium. (A) Measurements of Transepithelial electrical resistance (TEER) in RPMI 2650 cells RPMI 2650. Data are shown as mean ± SEM. (B) Optical observation of RPMI 2650 cells from the day of seeding up to 25 days in culture. Scale bar

500 µm. (C) Alcian Blue staining of RPMI 2650 cells to verify the presence of visible mucus. (D) Immunofluorescence staining for tight junctions marker (anti-occludin antibody, green; DAPI, blue) on RPMI 2650 grown on a cell insert membrane for 21 days. Scale bar 10 µm.

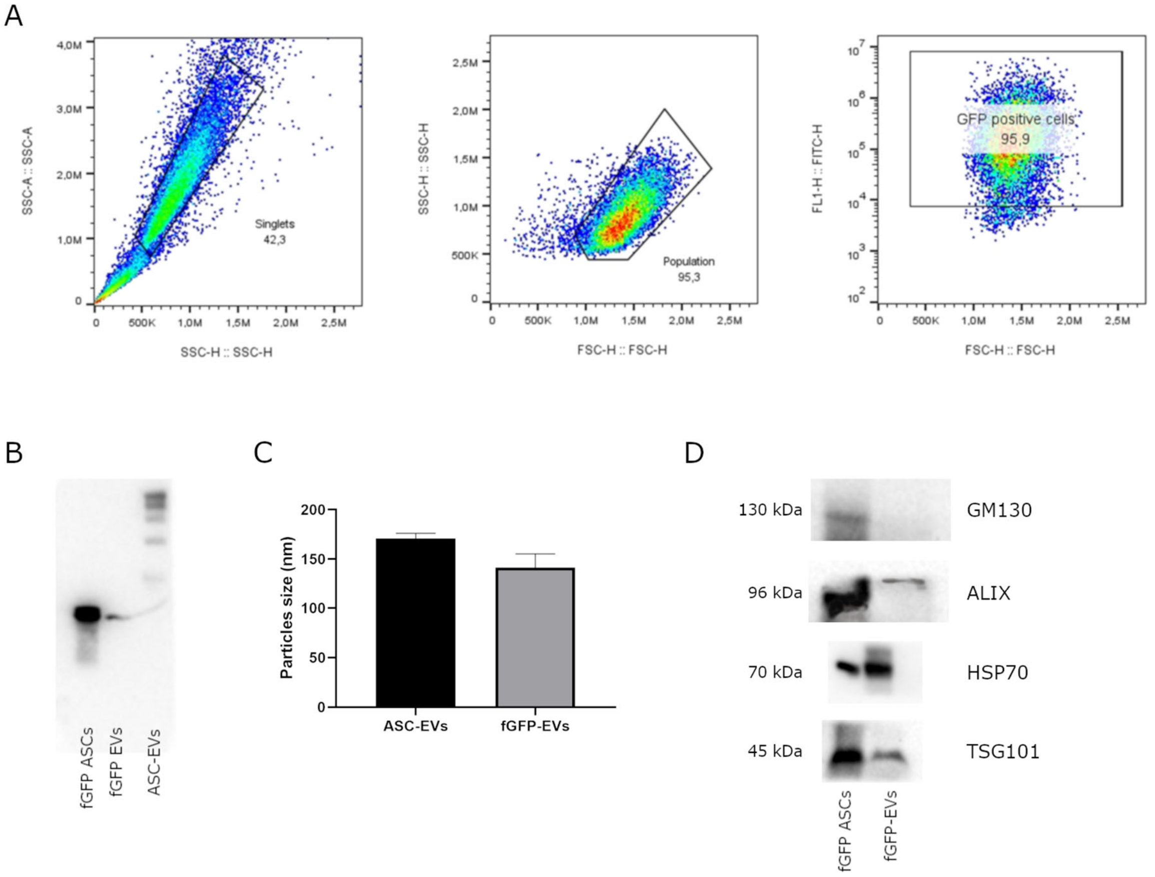

Figure S2. Characterization of fGFP-ASC and fGFP-EVs. (A) Representative dot plots showing the gating strategy to verify the purity of the sorted fGFP-ASCs positive cells. (B) Western blot analysis of fGFP expression in fGFP-EVs. fGFP-ASC cells and ASC-EVs derived from no-trasfected cells were used as positive and negative control respectively. (C) Particle size comparison between total extracted ASC-EVs and fGFP-EVs by NTA (n=5 measurements n.s.). Data are shown as mean ± SEM. (D) Markers expression in fGFP-EVs and in fGFP-ASC parental cells: ALIX (96 kDa), HSP70 (70 kDa) and TSG101 (45 kDa). GM130 (130 kDa) was used as negative control.

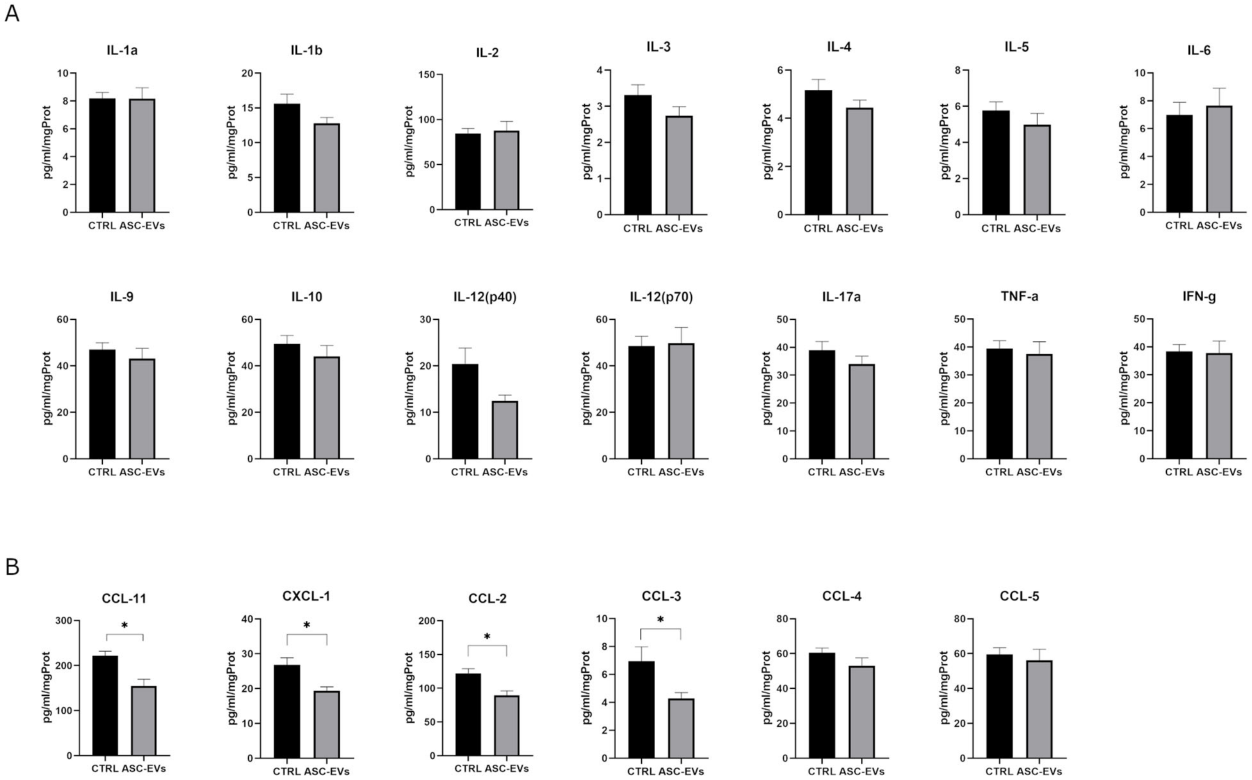

Figure S3. Cytokines and chemokines detection. Multiplex analysis of (A) cytokines and (B) chemokines detected in brain homogenates of EAE mice treated with ASC-EVs and PBS-injected controls. The protein concentration of the molecules is expressed as pg/ml/mg of the total protein content. Data are represented as the mean ± SEM of one representative experiment from a series of two similar results for n=5 mice/condition by Mann-Whitney test (*p<0.05).

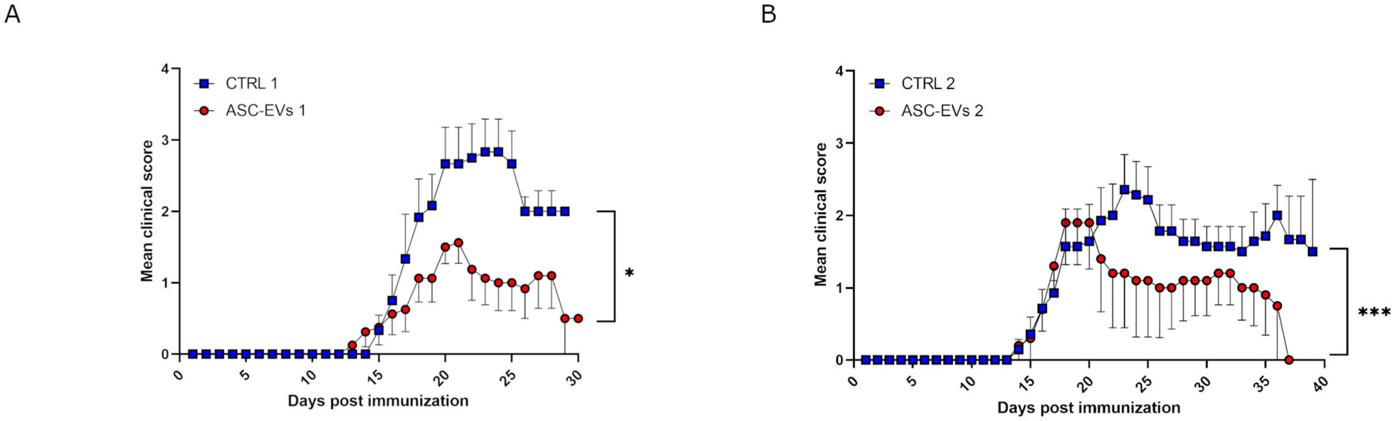

Disease parameters	CTLR	ASC-EVs treatment	P value
# animals	6	8	
Remission incidence	0/6	3/8	
Disease onset (d.p.i) (range)	16.67 ± 0.6667 (15-19)	17.38 ± 0.9808 (13-20)	n.s.
Maximum clinical score (range)	2.83 ± 0.4595 (2-4.5)	1.87 ± 0.2266 (1-3)	n.s.
Cumulative disease index (range)	26.50 ± 4.125* (19-42)	13.75 ± 3.155* (2.5-28.5)	* p = 0.0167

Disease parameters	CTLR	ASC-EVs treatment	P value
# animals	7	5	
Remission incidence	1/7	3/5	
Disease onset (d.p.i) (range)	17.14 ± 0.9863 (14-21)	16.00 ± 0.5477 (14-17)	n.s.
Maximum clinical score (range)	2.86 ± 0.3891 (1.5-4)	2.40 ± 0.5099 (1-4)	n.s.
Cumulative disease index (range)	37.43 ± 6.080 (9-52.5)	25.40 ± 11.09 (3-65)	n.s.

Figure S4. Clinical features of ASC-EVs daily i.n. administration in EAE mice. Clinical score and disease parameter of MOG35-55 EAE mice treated i.n. with PBS (vehicle) or with ASC-EVs of (A)

endpoint 1 and (B) endpoint 2. Data are represented as mean ± SEM. Two-tailed Kolmogorov-Smirnov test was used (*p<0.05, ***p<0.001). #= numbers of; n.s= not significant.

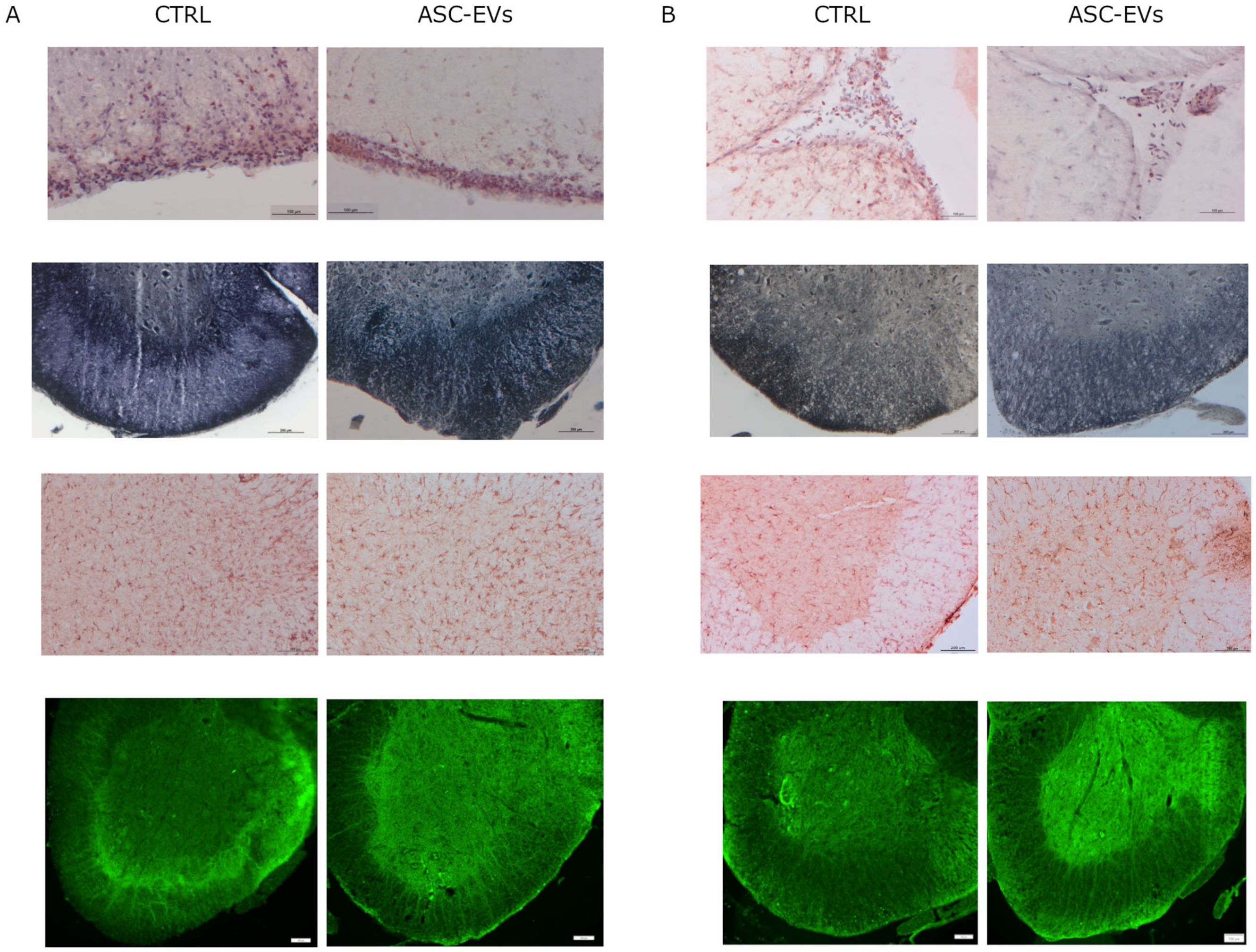

Figure S5. Lumbar spinal cord histology of ASC-EVs daily i.n. administration in EAE mice. Representative images of spinal cord sections for indicated markers. From the top to the bottom: CD3+ cells, demyelination (Woelcke staining), Iba-1+ cells and SMI-32 one day after the last administration (endpoint 1, A) or 10 days later (endpoint 2, B).
